# Early-career and fellow gynecologic oncologists perceive underpreparedness for the business of medicine: A Society of gynecologic oncology survey study

**DOI:** 10.1016/j.gore.2024.101501

**Published:** 2024-09-21

**Authors:** Jhalak Dholakia, Leslie R. Boyd, Rinki Agarwal, Haley Moss, Emily M. Ko, Emeline Aviki, Margaret I. Liang

**Affiliations:** a Division of Gynecologic Oncology, ECU Health, Greenville, NC, USA; b Division of Gynecologic Oncology, NYU Grossman School of Medicine, New York, NY, USA; c Division of Gynecologic Oncology, SUNY Upstate Medical University, Syracuse, NY, USA; d Division of Gynecologic Oncology, Department of Obstetrics and Gynecology, Duke Cancer Institute, Durham, NC, USA; e Division of Gynecologic Oncology, Department of Obstetrics and Gynecology, University of Pennsylvania, Philadelphia, PA, USA; f Division of Gynecologic Oncology, Department of Obstetrics & Gynecology, Cedars-Sinai Medical Center, Los Angeles, CA, USA

## Abstract

**Objective:**

There is a research gap on the impact of payment, reimbursement, and academic productivity in career decision-making for early-career (EC) attendings in gynecologic oncology. We sought to assess gynecologic oncology fellows and EC attendings on their knowledge and perceptions regarding the business of medicine.

**Methods:**

An anonymous survey was electronically disseminated to fellow and EC SGO members. Key themes were the business of medicine, productivity, and compensation/negotiation. A 5-point Likert scale was utilized; descriptive statistics were calculated using SPSS.

**Results:**

There was a 29 % response rate: 82 fellows and 102 EC attendings. Most were white (n = 143, 78 %) and female (n = 138, 75 %.) Most fellows (n = 67, 82 %) were interested in, and most EC (n = 82, 82 %) were employed in, academic/non-private practice. Fellows and EC attendings reported insufficient education on RVUs (relative value units) and reimbursement (80 %, n = 66; 81 %, n = 83) and did not feel prepared for the business aspect of practice (80 %, n = 66; 73 %, n = 75). Over 40 % of fellows did not understand how RVUs relate to practice. Thirty-three percent of EC attendings did not understand RVU assignments; 29 % were satisfied with methods used to determine productivity, and 17 % did not understand their compensation. Over 60 % of fellows felt unprepared to negotiate clinical productivity expectations. For EC attendings, 47 % were uncomfortable negotiating clinical expectations, 32 % negotiating academic expectations, and 52 % negotiating compensation changes. Female EC felt less prepared than male EC regarding the business of medicine (p = 0.02), RVU assignments (p < 0.01), and compensation negotiations (p < 0.01).

**Conclusion:**

Most gynecologic oncology fellows and early-career attendings do not feel prepared for the business of medicine. Women were less comfortable with these concepts than men. Formal education should be incorporated into career development curricula.

## Highlights

• Fellows and early-career gynecologic oncologists perceive insufficient education on the business of medicine.
• Women early in their careers are less comfortable with work RVUs and productivity concepts than their male colleagues.
• Almost 1 in 5 early-career gynecologic oncologists do not understand their compensation plan.

## 1 Introduction

Physician compensation models in medicine vary widely, with little transparency in their implementation. Compensation models involve expectations of clinical, research, and administrative productivity, as well as quality measures that are tied to physician compensation. The Society of Gynecologic Oncology (SGO) 2020 State of the Society Survey found that most gynecologic oncologists’ clinical income is determined by salary, with some bonus/incentive supplementation (State of the Society Survey., 2020). These bonuses are most often associated with productivity measured in work RVUs (relative value units). However, little is known about what gynecologic oncologists understand or desire in their career decision-making regarding reimbursement and productivity.

Compensation and career advancement concerns are major contributors to job dissatisfaction and turnover in the early-career period (Khaja, 2022, Saley, 2019, Kriss, 2021). A urology survey showed that most of their trainees and early graduates did not feel comfortable with the business aspect of practice, compensation, and contract negotiation (Atiemo, 2020). These concerns were reported as a primary reason for early-career job changes. Individual financial health and burnout concerns are particularly challenging for women and under-represented minorities in medicine, who tend to have lower compensation than their white, male counterparts (Verduzco-Gutierrez, 2021, Palepu, 2000). In urology, women were less comfortable with the business of medicine and expected to make a lower salary in the first year (Cone, 2021). Similar salary findings are noted by SGO: in 2020, base salaries in the first year of practice were almost $90,000 lower for women than for men. Mean income for URM gynecologic oncologists was $102,000 less than mean income for male respondents and $31,000 less than the overall mean income (State of the Society Survey., 2020). Furthermore, men with 1–10 years of experience were more likely to receive incentive-based bonuses than their female counterparts.

Female gynecologic oncologists are also disproportionately impacted by burnout (Davidson, 2022). Over 60 % of female US respondents feel overly stressed or that their life is unmanageable, compared to 39 % of male respondents (State of the Society Survey., 2020). There is a knowledge gap in causative factors, but the burden of burnout threatens a workforce with an increasing proportion of women entering the profession. Female OB/GYN subspecialists in academic medicine, including in gynecologic oncology, are significantly more likely than men to leave their original department within the early-career period (Rayburn, 2011). Black and URM OB/GYN faculty identified a lack of promotion, mentorship, and financial concerns as motivators to leave academia; similar motivations were reported in a survey of women oncologists (Merfeld, 2021, Abelson, 2018). Burnout has also been associated with lower compensation in women in the subspeciality of physical medicine and rehabilitation, suggesting a potentially vicious cycle (Verduzco-Gutierrez, 2021). If a poor foundational understanding of the business of medicine, reimbursement, and contract negotiation are contributing to these financial concerns and associated burnout, efforts to improve education may facilitate a stronger workforce. To address these concerns, we aimed to characterize the knowledge base and perceptions of gynecologic oncology fellows-in-training and early-career attendings regarding their contract negotiations and compensation.

## 2 Methods

This project was determined to be IRB-exempt by East Carolina University. An anonymous electronic survey was disseminated in March 2022 to SGO directory members who identified as either fellows or early-career gynecologic oncology attendings (up to 5 years post-fellowship graduation, identified through SGO membership registration records). Questions were based on similar surveys for urology trainees and early-career attendings (Atiemo, 2020, Cone, 2021). Potential participants were sent the survey three times via email and were presented with a QR code to access the survey at the SGO annual meeting held in March 2022. Demographic characteristics collected from participants included age, sex/gender, sexual orientation, marital status, role (fellow or early-career), geographic region, and location of primary practice. Questions were centered on three themes: the business of medicine and RVUs (Relative Value Units), academic productivity, and compensation/negotiation (Appendix 1.) Participant perceptions were measured using 5-point Likert scales. Responses were grouped into three groups for analysis: “strongly disagree/disagree,” “neutral,” or “strongly agree/agree.” Descriptive statistics, independent t-tests, and ANOVA analyses were performed using SPSS (IBM, IBM SPSS Statistics for Macintosh., 2021).

## 3 Results

### 3.1 Participant characteristics

There was a 29 % response rate, which included 82 fellow and 102 early-career respondents, from a total of 635 delivered surveys. Participant characteristics are summarized in Table 1. In both groups, most respondents were white (overall n = 143, 78 %) and female (overall n = 138, 75 %). Most respondents identified as straight/heterosexual (n = 161, 88 %) and married (n = 128, 70 %). Geographic region representation was overall well-distributed. Most fellows and EC attendings practiced in urban settings.

**Table 1:** Demographics.

	Fellows N=82	Early-Career N=102
	N, %	N, %
**Race/Ethnicity**		
Asian American/Pacific Islander	14 (17)	11 (10)
Black/African American	4 (5)	8 (8)
Hispanic/Latino	6 (7)	4 (4)
White	61 (74)	81 (80)

**Sex/Gender**		
Female	62 (76)	76 (75)
Male	19 (23)	24 (25)

**Sexual Orientation**		
Straight/Heterosexual	71 (87)	90 (89)
Gay/Lesbian	7 (9)	4 (4)
Bisexual	2 (2)	5 (5)
Queer	1 (1)	0
Other/Prefer not to answer	1 (1)	2 (2)

**Marital Status**		
Married	44 (54)	84 (84)
Single, never married	21 (26)	9 (9)
Single but cohabiting with significant other	14 (17)	6 (5)
Legal domestic partnership/civil union	1 (1)	0
Divorced	0	1 (1)
Other/prefer not to answer	2 (2)	1 (1)

**Geographic Region**		
Midwest	16 (20)	27 (27)
Northeast	26 (32)	16 (16)
South	25 (30)	43 (43)
West	14 (17)	14 (13)
Prefer not to answer	1 (1)	1 (1)

**Practice Location**		
Urban	60 (73)	59 (58)
Suburban	6 (7)	21 (21)
Small/Medium Cities	14 (17)	21 (21)
Rural	1 (1)	0
Prefer not to answer	1 (1)	0

### 3.2 Factors influencing practice type/preference

**Fig. 1:**
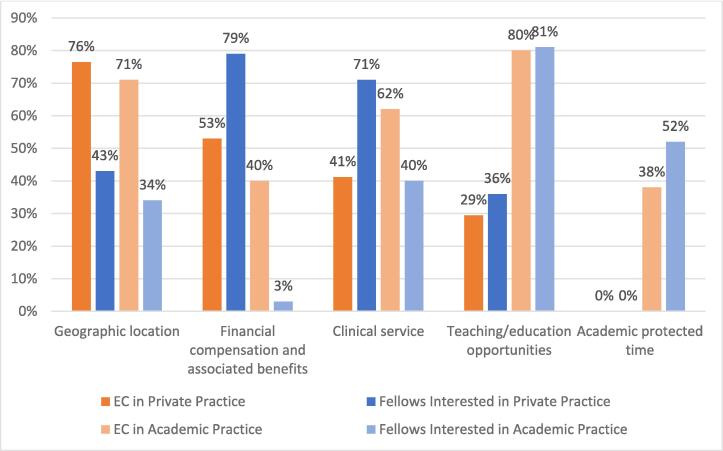
Factors Influencing Practice Type/Intended Practice Type.

The majority of EC attendings were in non-private/academic practice (n = 82, 82 %), with a similar proportion of fellows interested in academic practice employment after graduation (n = 67, 82 %). Both groups were asked to select from a checklist of “reasons why you chose this practice type,” and respondents could select multiple options. Teaching/education opportunities were the most common factor associated with academic practice for fellows and EC attendings (81 % and 80 %, respectively). Financial compensation and benefits were the predominant factor for the fellows (79 %) interested in private practice. Geographic location was also a predominant factor for early-career in private practice (76 %) and early-career in academic practice (71 %) (Fig. 1.).

### 3.3 Perceptions and understanding of the business of medicine and RVUs (Relative value Units)

**Fig. 2:**
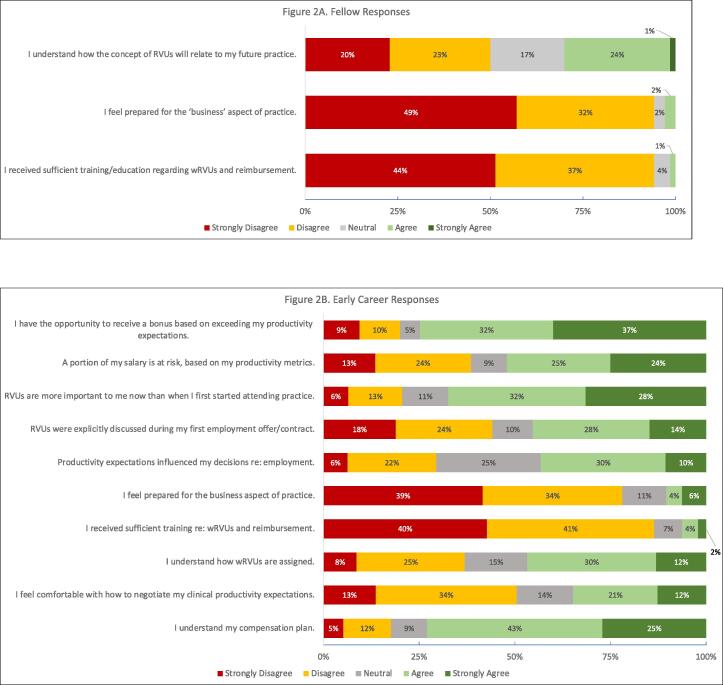
Fellow and Early Career Responses regarding Concept 1, the Business of Medicine and RVUs.

The majority of fellows and EC attendings did not feel prepared for the business of medicine (“strongly disagree/disagree,” fellows = 81 %, EC=73 %.) 78 % of fellows and 81 % of EC attendings did not believe that they received sufficient education on wRVUs (work RVUs, the component of RVUs related to physician work) and reimbursement. In the early-career group, 42 % reported that wRVU expectations were discussed during their first employment offer, and over half (60 %) agreed that RVUs were more important to them now than when they started attending practice. One-third of EC attendings did not understand how wRVUs are assigned, and only 29 % were satisfied with the methods used to determine productivity metrics. 17 % of EC attendings responded “disagree/strongly disagree” to the statement “I understand my compensation plan.” (Fig. 2.).

### 3.4 Perceptions and understanding of academic productivity

**Fig. 3:**
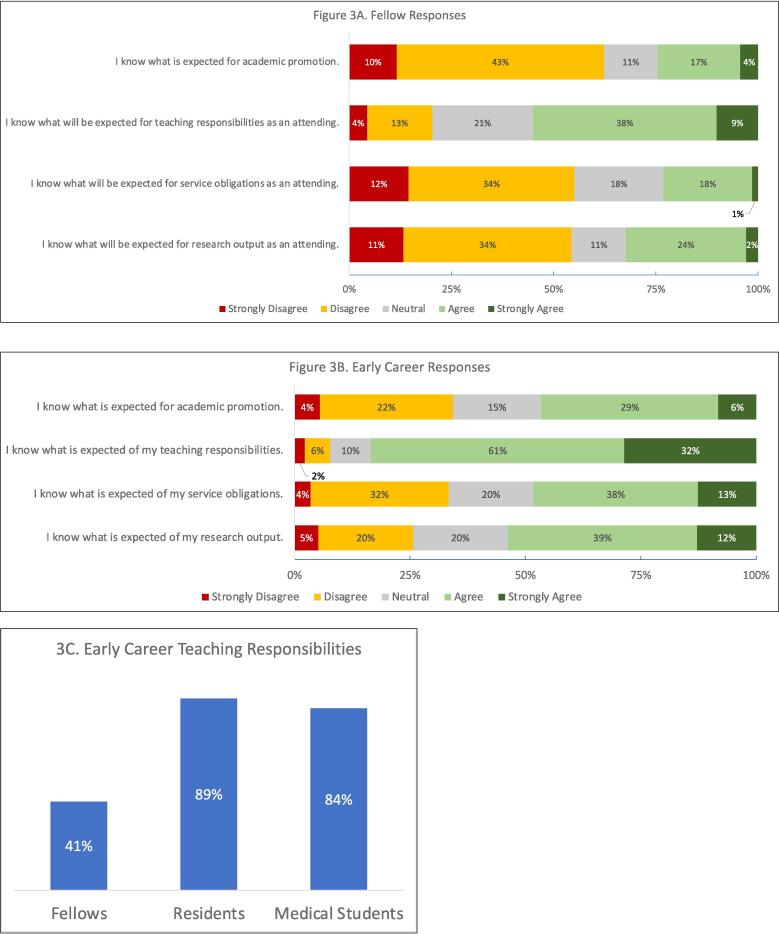
Responses Re: Concept 2, Academic Productivity.

We also assessed understanding of productivity expectations as an attending (Fig. 3.) Despite 82 % of fellows hoping to enter academic practice, few understood what to expect for the service and research components of the academic triad (19 % and 26 %, respectively.) A higher proportion of fellows agreed that they understood teaching expectations (47 %.) Over half of fellows did not know what was expected for academic promotion. Most EC attendings knew their teaching expectations (93 %), and most were responsible for resident and medical student education (Fig. 3C.) Fewer understood their service and research expectations (51 % for both.) One-quarter of EC attendings did not know what was expected for academic promotion.

### 3.5 Perceptions and understanding of contracts and negotiation

**Fig. 4:**
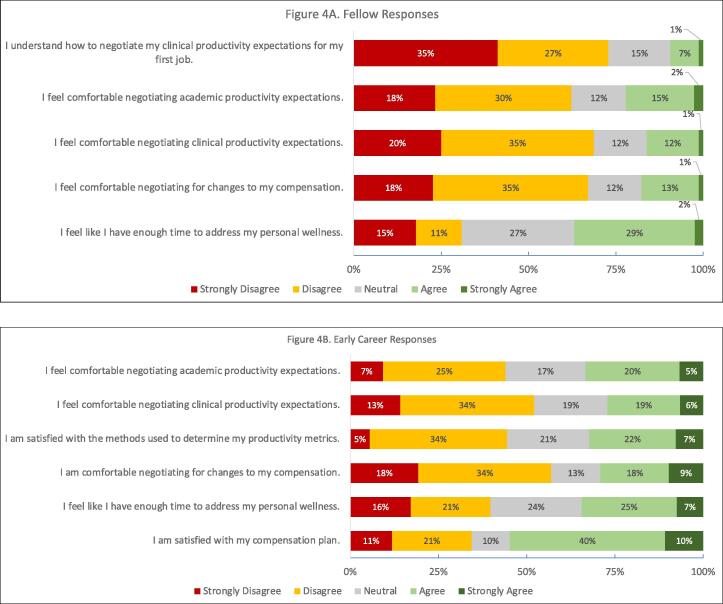

Most fellows and EC attendings (53 %, 52 %) were uncomfortable negotiating for changes to their compensation (Fig. 4.) 55 % of fellows and 47 % of EC attendings did not feel comfortable negotiating for changes to clinical productivity. 48 % of fellows and 32 % of EC attendings felt uncomfortable negotiating for changes to academic productivity. 62 % of fellows did not understand how to negotiate clinical productivity expectations for their first job.

### 3.6 Differences in response based on gender

**Fig. 5:**
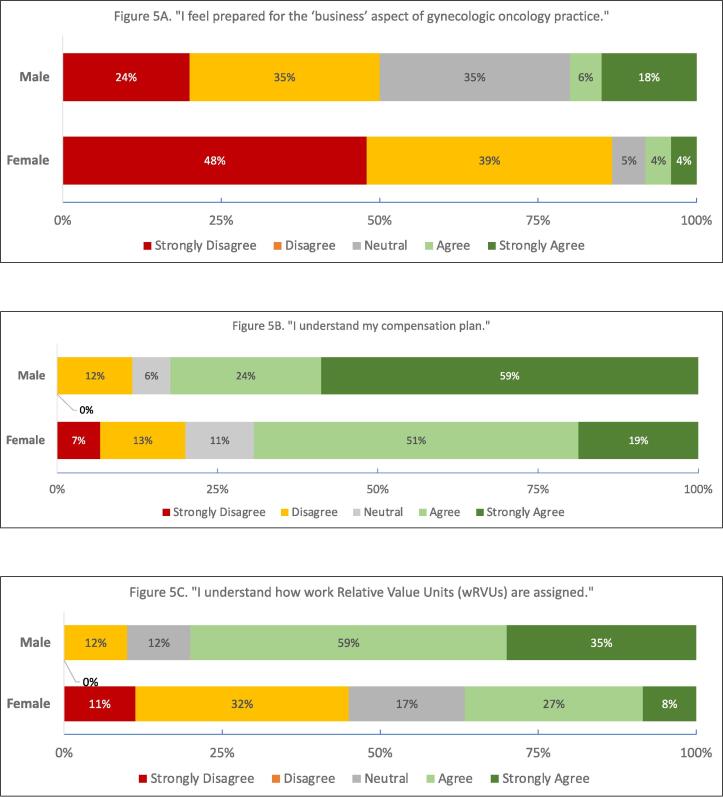

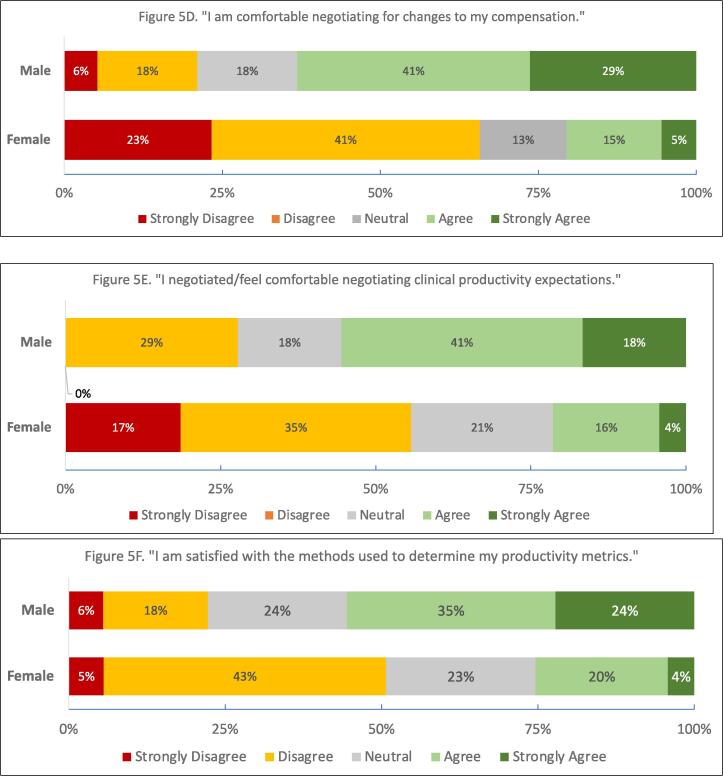
Gender Differences in Early-Career Responses (p < 0.05 male vs female, all.).

Significantly fewer EC women felt comfortable with the business of medicine than their male colleagues (p = 0.02, Fig. 5A); this difference was not noted in the fellows group. EC women disagreed more than men with the statement “I understand my compensation plan” (p < 0.01, Fig. 5B.) Fewer early-career women reported that they understood how wRVUs were assigned (p < 0.01, Fig. 5C.) With respect to negotiation, EC women felt less comfortable negotiating for changes to compensation and clinical productivity (p < 0.01, Fig. 5D, 5E.) EC women more often reported dissatisfaction with the methods to determine their productivity (p = 0.03, Fig. 5F.) These proportions were similar in private vs academic practice. No sex/gender differences were noted in the fellows group for any question.

## 4 Discussion

This survey is the first to assess attitudes regarding the business of medicine, compensation, and negotiation in gynecologic oncology trainees and early-career attendings. Our findings demonstrate that despite recognition that compensation and professional development are important components of career planning, nearly all gynecologic oncology fellows (81 %) and early-career attendings (71 %) feel underprepared for this important aspect of medical practice.

Most fellows and EC attendings reported insufficient education regarding RVUs and the business of medicine. This, taken with the fact that 60 % of EC attendings reported that RVUs were more important to them after starting practice, suggests an under-preparedness for understanding first contracts. Over 60 % of gynecologic oncology fellows in this survey did not understand how to negotiate productivity expectations for their first job, and the majority do not understand expectations for the service and research components of academic medicine.

Notably, there were significant differences between male and female early-career respondents. Early-career women were less comfortable with the concept of the business of medicine and expressed more dissatisfaction with their methods of productivity assessment and compensation. This relates to potential trends in gender disparities noted in other studies. The Society of Gynecologic Oncology (SGO) 2020 State of the Society survey found that men and women earn the same dollar amount per wRVU. However, men reported significantly higher annual wRVUs (medians, 7500 vs 5500), which translated to higher personal income for men. Men were also more likely to receive bonus payments, often associated with wRVU-based productivity. This was most pronounced for respondents in the first 10 years of practice (State of the Society Survey., 2020). A study looking at a single OB/GYN department found that although total productivity and salaries were similar between male and female general OB/GYN faculty, the type of care that contributed to these values differed between groups. Female faculty saw more new patients, but male providers had higher wRVU per encounter (Zhang, 2019). EC women in our study were less comfortable with metrics that may impact these calculations: explicitly, how wRVUs are assigned and how productivity is calculated. These data suggest that the difference may not lie in different objective reimbursement rates per unit of labor but in how providers understand and utilize the revenue-generating components of their care such as billing and productivity assessment. Financial health and perceptions of compensation are associated with burnout, which also disproportionately impacts women. In the 2020 SGO survey, women reported worse work-life balance than men, and a follow-up survey found that over 60 % of female gynecologic oncologists experienced symptoms on the Maslach Burnout Inventory (Davidson, 2022). Burnout represents a threat to the individual as well as the workforce, ultimately risking the care for individuals with gynecologic malignancies. Knowledge gaps regarding the business of medicine thereby represent a potential opportunity to mitigate causes of burnout, particularly for women.

Poor understanding of compensation and negotiation also disadvantages new graduates when planning career advancement, both in academics and private practice. Our findings demonstrated that most fellows planned to work in academics. However, most did not understand expectations for service and research as part of the professional academic triad. Preparing trainees for productivity expectations and negotiation at the outset of their careers may represent an area of growth for the organization and its members.

Limitations of our study include the survey nature of assessing respondent attitudes, which may be more complex than what is captured in a Likert scale. There is a potential for selection bias: people who are more interested in the topics of productivity and reimbursement or those with strong feelings about the topics may have been more likely to respond. Similarly, only 18 % of respondents were interested or employed in non-academic/private practice models: a comparison of these individuals with their academic counterparts may reveal differences in self-selection for practice type and participation in surveys and programs related to the business of medicine. We acknowledge that the 29 % response rate introduces potential non-responder bias, and hope that future efforts in this space, including qualitative interviews, may provide additional information to better inform professional development programs. A small number of under-represented minority (URM) respondents limits our ability to interpret for this group (Verduzco-Gutierrez, 2021). Efforts to promote URM in gynecologic oncology are ongoing, including at the trainee level; incorporating business of medicine education may be beneficial in such programs.

Strengths of this study include that 29 % of all fellows and early-career attendings in SGO participated in the survey, which was administered electronically over a short time period around the 2022 SGO Annual Meeting. This suggests that content related to the business of medicine is of interest to this membership and indicates that young members will engage with associated efforts. Demographics were similar to those reported in the 2020 SGO State of the Society survey, underscoring the representative sample reached in this effort. Potential action items may include incorporating the business of medicine into the fellowship training curriculum, developing formal medical society early-career education programs, and developing a mentorship program for EC gynecologic oncologists interested in advocating for the business of medicine. Further efforts in this area merit investigation and support.

## 5 Conclusion

This study demonstrates an alarming knowledge and training gap regarding the business of medicine for gynecologic oncology trainees and early-career attendings. This gap has potential implications on financial health, burnout, and long-term career success and may disproportionately impact women in our specialty. Programs to address these professional development needs may ultimately strengthen the gynecologic oncology workforce and contribute to the specialty's strength, facilitating the overall goal of improved care for individuals with gynecologic malignancies.

## CRediT authorship contribution statement

**Jhalak Dholakia:** Writing – review & editing, Writing – original draft, Validation, Methodology, Formal analysis, Data curation, Conceptualization. **Leslie R. Boyd:** Writing – review & editing, Validation, Supervision, Project administration, Methodology, Conceptualization. **Rinki Agarwal:** Writing – review & editing, Methodology, Conceptualization. **Haley Moss:** Writing – review & editing, Methodology, Conceptualization. **Emily M. Ko:** Writing – review & editing, Supervision, Methodology, Conceptualization. **Emeline Aviki:** Writing – review & editing, Methodology, Conceptualization. **Margaret I. Liang:** Writing – review & editing, Methodology, Conceptualization.

## Appendix 1

**Understanding payment structures in gynecologic oncology**.

The way we value work is a critically important topic for all our SGO members, yet there is almost no transparency on how various systems are used, particularly for those in the early stage of their career. This has taken particular importance with the recent findings of the State of the Subspecialty survey which demonstrate inequity based on gender. Moreover, optimizing payment structures has the potential to improve the quality of cancer care delivered and improve provider satisfaction/reduce burnout.

The goal of this survey is to obtain perspectives from trainees and early career gynecologic oncologists on payment structures in gynecologic oncology. We appreciate your time and responses to this survey. This survey is considered IRB exempt by New York University.

Demographics.

1. Age group
  a. <30y
  b. 30–39
  c. 40–49
  d. 50–59
  e. 60–69
  f. ≥70
2. Race/Ethnicity (select all that apply)
a. White
b. Asian American or Pacific Islander
c. Black or African American
d. Hispanic/Latino
e. American Indian/Alaska Native
f. Other (please specify)
3. Sex/Gender
a. Male
b. Female
c. Non-binary
d. Other (please specify)
4. Sexual Orientation
a. Asexual
b. Bisexual
c. Gay or Lesbian
d. Straight/Heterosexual
e. Queer
f. Other (please specify)
5. Marital Status
a. Married
b. Widowed
c. Divorced
d. Separated
e. Legal domestic partnership/civil union
f. Single but cohabiting with significant other
g. Single, never married
h. Other (please specify)
6. What is your role?
a. Fellow (please provide year in text box below)
b. <1 year
c. 1–5 years in practice
d. 6–10 years in practice
e. Fellowship PGY (if applicable)
7. Early Career Only Question: How many post-fellowship jobs have you had?
8. Early Career Only Question: Which employment status best describes your practice type
a. Private Practice
b. Non-Private Practice
9. Fellow Only Question: Which employment status best describes the type you are most interested in?
a. Private Practice
b. Non-Private Practice
10. Early Career Only Question: If in Private Practice, which employment status best further describes your practice type
a. Owner − Solo practice
b. Partner − Single specialty partnership or group
c. Partner − Multi-specialty partnership or group
d. Employee or “junior partner” of a private practice
11. Fellow Only Question: If Private Practice, which employment status best further describes the type you are most interested in
a. Owner − Solo practice
b. Partner − Single specialty partnership or group
c. Partner − Multi-specialty partnership or group
d. Employee or “junior partner” of a private practice
12. Early Career Only Question: If in Non-Private Practice, which employment status best further describes your practice type
a. University or medical school
b. Private hospital
c. Multispecialty clinic
d. Health maintenance organization (HMO)
13. Federal, State or local government
46. Fellow Only Question: If Non-Private Practice, which employment status best further describes the type you are most interested in
  a. University or medical school
  b. Private hospital
  c. Multispecialty clinic
  d. Health maintenance organization (HMO)
  e. Federal, State or local government
14. Why did you chose this practice type? (select all that apply)
a. Academic protected time
b. Administrative leadership opportunities
c. Career advancement in training programs
d. Clinical outreach sites
e. Geographic location
f. Public Service Loan Forgiveness (PSLF)/loan repayment
g. Research protected time or resources
h. Clinical trials
i. Ownership benefits
j. Teaching/education opportunities
k. Clinical service
l. Financial compensation and associated benefits
m. Other (please specify)
15. Early Career Only Question: Clinical and non-clinical breakdown (select all that apply)
a. I have protected administration time
b. I have protected academic time for research or teaching or other
16. Early Career Only Question: Percentage of time spent on: choices (N/A, 0–20 %, 21–40 %, 41–60 %, 61–80 %, 81–100 %)
a. Clinical
b. Administration
c. Research
d. Teaching
e. Other
17. Geographic region
a. Northeast
b. Midwest
c. South
d. West
18. Location of Primary Practice
a. Suburban
b. Rural
c. Urban
d. Small/Medium Cities

All below questions notated (L) were Likert scale format with the following options:

• N/A
• Strongly disagree
• Disagree
• Neutral
• Agree
• Strongly agree

Concept 1: The Business of Medicine and RVUs (Relative Value Units).

19. I received sufficient training/education regarding wRVUs and reimbursement (L)
20. I feel prepared for the ‘business’ aspect of gynecologic oncology practice (L)
21. Fellow Only Question: I understand how the concept of RVUs will relate to my future practice (L)
22. Early Career Only Question: I understand my compensation plan (L)
23. Early Career Only Question: I feel comfortable with how to negotiate my clinical productivity expectations (L)
24. Early Career Only Question: I understand how work Relative Value Units (wRVUs) are assigned (L)
25. Early Career Only Question: Productivity expectations influenced my decisions regarding employment (L)
26. Early Career Only Question: RVUs were explicitly discussed during my first employment offer/contract (L)
27. Early Career Only Question: RVUs are more important to me now than when I first started attending practice (L)
28. Early Career Only Question: A portion of my salary is at risk, based on my productivity metrics (L)
29. Early Career Only Question: I have the opportunity to receive a bonus based on exceeding my productivity expectations (L)

Concept 2 Academic Productivity:

30. Fellow Only Question: I know what will be expected for research output as an attending (i.e., publications, abstracts, presentations, grants) (L)
31. Fellow Only Question: I know what will be expected for service obligations as an attending (i.e., committees, advocacy, community outreach/volunteer work) (L)
32. Fellow Only Question: I know what will be expected for teaching responsibilities as an attending (i.e., medical student, resident, and fellow education) (L)
33. Early Career Only Question: I know what is expected of my research output (i.e., publications, abstracts, presentations, grants) (L)
34. Early Career Only Question: I know what is expected of my service obligations (i.e., committees, advocacy, community outreach/volunteer work) (L)
35. Early Career Only Question: I know what is expected of my teaching responsibilities (i.e., medical student, resident, and fellow education) (L)
36. Early Career Only Question: My formal teaching responsibilities include (select all that apply)
a. Fellow education
b. Medical student education
c. Resident education
37. I know what is expected for academic promotion (L)
38. My institution uses a discrete academic output metrics system (such as academic RVUs or an academic scorecard)
d. Yes
e. No
f. Don’t know

Concept 3: Compensation/Negotiation

39. I feel like I have enough time to address my personal wellness (L)
40. I am comfortable negotiating for changes to my compensation (L)
41. I negotiated/feel comfortable negotiating clinical productivity expectations (L)
42. I negotiated/feel comfortable negotiating academic (i.e., research, service, education/teaching) productivity expectations (L)
43. Fellow Only Question: I understand how to negotiate my clinical productivity expectations for my first job (L)
44. Early Career Only Question: I am satisfied with my compensation plan (L)
45. Early Career Only Question: I am satisfied with the methods used to determine my productivity metrics (L)
